# Biological Soil Crust From Mesic Forests Promote a Specific Bacteria Community

**DOI:** 10.3389/fmicb.2022.769767

**Published:** 2022-03-16

**Authors:** Karin Glaser, Martin Albrecht, Karen Baumann, Jörg Overmann, Johannes Sikorski

**Affiliations:** ^1^Applied Ecology and Phycology, Institute for Biological Sciences, University of Rostock, Rostock, Germany; ^2^Department of Soil Science, Faculty of Agricultural and Environmental Science, University of Rostock, Rostock, Germany; ^3^Department of Microbiology, Faculty of Life Sciences, Technische Universität Braunschweig, Braunschweig, Germany; ^4^Leibniz-Institute DSMZ, German Collection of Microorganisms and Cell Cultures, Braunschweig, Germany

**Keywords:** microbial interaction, biofilms, biological soil crusts, forest soil, terrestrial algae, biodiversity

## Abstract

Biological soil crusts (biocrusts) harbor a diverse community of various microorganisms with microalgae as primary producers and bacteria living in close association. In mesic regions, biocrusts emerge rapidly on disturbed surface soil in forest, typically after clear-cut or windfall. It is unclear whether the bacterial community in biocrusts is similar to the community of the surrounding soil or if biocrust formation promotes a specific bacterial community. Also, many of the interactions between bacteria and algae in biocrusts are largely unknown. Through high-throughput-sequencing analysis of the bacterial community composition, correlated drivers, and the interpretation of biological interactions in a biocrust of a forest ecosystem, we show that the bacterial community in the biocrust represents a subset of the community of the neighboring soil. Bacterial families connected with degradation of large carbon molecules, like cellulose and chitin, and the bacterivore *Bdellovibrio* were more abundant in the biocrust compared to bulk soil. This points to a closer interaction and nutrient recycling in the biocrust compared to bulk soil. Furthermore, the bacterial richness was positively correlated with the content of mucilage producing algae. The bacteria likely profit from the mucilage sheaths of the algae, either as a carbon source or protectant from grazing or desiccation. Comparative sequence analyses revealed pronounced differences between the biocrust bacterial microbiome. It seems that the bacterial community of the biocrust is recruited from the local soil, resulting in specific bacterial communities in different geographic regions.

## Introduction

Biological soil crusts (biocrusts) make up 12% of terrestrial ground cover and over 50% in both cold and hot arid regions where vascular plants cannot thrive (Belnap et al., 2016; Borchhardt et al., 2017). They also act as pioneer stages in soil recolonization, e.g., after disturbance (Belnap et al., 2003). Biocrusts are an association of microorganisms that strongly interact with the surrounding soil thereby shaping biogeochemical and physical properties of the first millimeters of the soil surface. Biocrusts consist of a huge variety of organisms, such as bacteria, microfungi, microalgae, lichens, and mosses. These organisms live on and interact with the underlying soil and the atmosphere in terms of nutrient cycling, water-holding capacity, and soil stabilization. Algal and cyanobacterial filaments, fungal hyphae, and their extracellular polymeric substances glue soil particles together, which results in a stable biocrust (Belnap et al., 2016). Furthermore, nutrients such as carbon, nitrogen, and phosphorus are enriched in biocrusts (Beraldi-Campesi et al., 2009; Dümig et al., 2014; Baumann et al., 2017). Prokaryotic nitrogen fixation at the soil surface can even result in a net transfer of inorganic plant available nitrogen into the underlying soil (Johnson et al., 2007). Also the bacterial nitrogen turnover is enhanced in biocrusts compared to bare soil or underlying soil (Brankatschk et al., 2013). Thus, biocrusts represent microecosystems with intense interactions between autotrophic and heterotrophic components of both prokaryotic and eukaryotic organisms.

Many previous studies focused on the persistent biocrusts of extreme habitats, their prokaryotic and eukaryotic diversity (Büdel et al., 2016), ecophysiology of isolated strains (Gray et al., 2007; Karsten et al., 2010), net primary productivity *in situ*, or their development and succession as the dominant vegetation form in these areas (Belnap et al., 2016). In the temperate zone, particularly extreme weather events as drought or storms affect the vegetation also in temperate forests (Schelhaas et al., 2003) and create open spaces for biocrust development (Belnap et al., 2001). Bare soil at high vegetation climax sites is generated by animal activities and windfall, and by forest management, such as the removal of trees and the creation of skid trails or driving lanes for heavy equipment. The favorable climatic conditions in middle European temperate regions allow biocrusts to develop within weeks after disturbance, usually starting with a biofilm of eukaryotic algae, which are, besides mosses, the most dominant biocrust phototrophs in temperate forests and heaths (Gypser et al., 2015; Glaser et al., 2017, 2018). Biocrusts in temperate regions can sustain many years, but their activity is seasonal dependent (Dümig et al., 2014; Rieser et al., 2021). Even in well-developed European forest soils, biocrusts accumulate nutrients more strongly than surrounding bulk soils (Baumann et al., 2017; Drahorad et al., 2020) and also might counteract soil erosion as has been shown in other forest ecosystems (Seitz et al., 2017). Furthermore, temperate biocrusts impact the hydrological characteristics of the respective habitat (Gypser et al., 2016b).

Cyanobacteria and eukaryotic microalgae are crucial for the biocrust formation and development as major contributors to carbon fixation (Büdel et al., 2016; Szyja et al., 2018). Eukaryotic algae are the least studied phototrophs in biocrusts, even though as many as 350 species have been described in biocrusts to date (Büdel et al., 2016). Eukaryotic algae play minor roles in the formation of biocrusts in hot arid regions compared to cyanobacteria (Büdel et al., 2016) but are found more prominently in Arctic and Antarctic regions (Belnap et al., 2016; Pushkareva et al., 2016; Rippin et al., 2018a) as well as in Mediterranean and temperate climates (Glaser et al., 2017, 2018; Samolov et al., 2020). For temperate biocrusts, hardly anything is known about the role of microalgae and their interaction with the environment or surrounding microbiome (Corbin and Thiet, 2020), even though biocrust can reach locally high coverage rates as determined by remote sensing for example in sandy heaths (Rieser et al., 2021).

With the exception of cyanobacteria, biocrust bacterial communities only recently gained increasing attention by applying high-throughput-sequencing analyses. Archaea, Chloroflexi, and anaerobic bacteria appeared to be less prominent in the biocrusts than below the biocrust (Steven et al., 2013). Data on the bacterial community in biocrusts and underlying soils of the same sampling site are so far restricted to semiarid sites (Steven et al., 2013; Albright et al., 2019; Moreira-Grez et al., 2019; Pombubpa et al., 2020). These studies at different arid geographical regions, Western Australia, Colorado Plateau, and the Mojave Desert, United States, found major differences in the bacterial communities between biocrusts and underlying soils. Cyanobacteria were enriched in the biocrusts, whereas bacterial richness was higher in the underlying soils for most bacterial and archaea groups. The same pattern was found for bare surface soils in close proximity to the biocrust covers, which appear to have a higher microbial diversity than the biocrusts (Abed et al., 2019; Moreira-Grez et al., 2019). Compared to bare soil, biocrusts promoted high proportions of Proteobacteria and Acidobacteria in Western Australia (Moreira-Grez et al., 2019), whereas in Oman, Actinobacteria were reported as the most abundant bacteria phylum in most biocrust samples (Abed et al., 2019). It seemed that biocrusts affect the bacterial diversity by promoting a smaller subset of the bulk soil microbiome (Moreira-Grez et al., 2019). For these biocrusts, network analyses revealed most connections in bacteria–bacteria interactions rather than cross-domain interactions of bacteria and fungi, both in the biocrust and underlying soil alike (Pombubpa et al., 2020).

Algae–bacteria interactions are known from different habitats and even found their way into biotechnological applications [reviewed in Ramanan (2016)]. So far, only Rippin et al. (2018b) studied eukaryotic microalgae and their interaction with the bacterial communities in Arctic and Antarctic biocrusts by analysis of co-occurrence networks. These networks revealed clusters of multiple nodes combining eukaryotic and prokaryotic organisms indicating cross-domain interactions. Close interactions of algae and bacteria in biocrusts are very likely because exopolysaccharides and decaying biomass of the phototrophic community provide organic material for the growth of the heterotrophic community. Previous studies reported an altered bacterial community depending on the biocrust developmental stage, which was determined by the dominating phototrophic organisms (Maier et al., 2018; Miralles et al., 2021). The identity of the main phototrophic species likely influences the biocrust microbiome since microalgae species differ in their functional traits such as excretion of exopolysaccharides (EPS). Those EPS protect the microalgae from predation or desiccation but also shape the soil matrix and could serve as a carbon source for the heterotrophic community. During biocrust development on organic-poor soils, also bacterial EPS genes become more abundant (Cania et al., 2019). The influence of the biocrust phototrophs on the bacterial community has been addressed for poorly developed semiarid and Arctic soils (Maier et al., 2018; Juottonen et al., 2020) but not for well-developed soils as they occur in temperate forests. Only few studies were conducted so far in temperate regions with open landscapes like heaths or postmining areas studying the nutrient turnover in biocrusts at different developmental stages (Brankatschk et al., 2013; Dümig et al., 2014; Gypser et al., 2016a). It may be expected that the organic-rich temperate forest soils may support a rich community of heterotrophic bacteria, which are less dependent on organic input by microalgae.

We expect that the functional traits of algae, such as a filamentous life form or EPS excretion, affect the bacterial diversity. Comparing biocrusts and bare soil, we hypothesize that the bacteria in biocrusts are a subset of the overall soil bacteria favoring specialists, such as saprotrophic bacteria. Furthermore, we analyzed the factors driving bacterial and algal community in German forest biocrusts and considering environmental parameters such as soil properties, main tree species, and forest management.

## Materials and Methods

### Study Site and Sampling

Samples were taken from German forest plots of the Biodiversity Exploratories, in Swabian Alb (a low-mountain site), Hainich (a lowland), and Schorfheide-Chorin (moraine landscape) (Fischer et al., 2010). The forests are dominated by deciduous (European beech *Fagus sylvatica*) or coniferous species (Scots Pine *Pinus sylvestris* or Norway spruce *Picea abies*). The plots were either located in natural protected forest areas or in managed forests (age-class) and were characterized by their silvicultural management intensity index (SMI) and the effect of management on the stand density (SMId). The numerical index SMI takes into account the SMId, main tree species, and stand age and ranges between 0 and 1 (Schall and Ammer, 2013). The higher the SMI, the more intense the management. The higher the SMId, the more intense the effect of the silvicultural management on the natural stand density. The SMId ranged in our sampling site from 0 in the natural forests to 0.57 in an intensively managed beech forest.

Biological soil crusts were collected in June 2014 and 2015 from litter-free bare soil in the forests like on root plates of fallen trees, in digging holes of wild boars, and at exposed mounds at all three sampling sites. Biocrusts were visible by eye as green cover without lichen or moss thalli. For the identification of algae, the top soil, on which biocrusts had been visually detected as green cover, was collected by pressing the opening of a petri dish into the biocrust and removing it gently with a spatula. After transportation to the lab, the upper two millimeters of the biocrust was separated from the adhering soil underneath using a razor blade and stored air-dry in paper bags prior to cultivation. For elemental analyses, samples were finely ground using a mortar grinder (Pulverisette 2, Fritsch, Idar-Oberstein, Germany). Samples used for DNA extraction were collected next to the previous sampling spot. For this, a small (5 mm) cork borer was used to transfer the top 2 mm of soil in a plastic tube, which was immediately frozen in the field at –80°C.

Bulk soil samples were collected during the sampling campaign in May 2014 following the procedure described earlier (Solly et al., 2014). Samples were immediately frozen at –80°C for further analyses.

### Element Content in Biocrusts and Soils

The total C, N, and S contents of biocrusts were determined by dry combustion using an elemental analyzer (VARIO EL, Elementar Analysensysteme GmbH, Hanau, Germany). Total P content was measured after microwave-assisted *aqua regia* digestion using inductively coupled plasma optical emission spectroscopy (ICP OES) (JY 238 UL Trace, France). All analyses were done in duplicate.

### Cultivation, Identification, and Richness of Algae

The procedure for culturing (enrichment cultivation) and morphological identification of algae and cyanobacteria followed exactly the procedure described earlier (Glaser et al., 2018). The results of one experimental site (Schorfheide) were already published. Here, we extent the data set by two more study sites (Swabian Alb and Hainich) and compare the algae with the bacterial community.

We used the total number of algae and cyanobacteria species per sample, i.e., species richness, as the measure of alpha diversity. As a measure of beta diversity, the similarity between the plots was calculated based on the presence/absence of individual species using the Bray–Curtis dissimilarity index, combining the total number and the identity of all algal taxa observed. As the ecological functions of individual species differ, the identified algae and cyanobacteria were categorized based on (1) their life form (filamentous or coccal), (2) characteristics of mucilage (no mucilage, mucilage only around individual cells, or strong mucilage around colonies), and (3) organism size (pico < 20 μm, nano > 20 μm and < 200 μm, micro > 200 μm).

### DNA Extraction, High-Throughput Sequencing, and Taxonomic Classification of Marker-Gene Amplicons

For bacterial community analyses, DNA was extracted from the biocrusts using a MoBio Soil Extraction Kit (MoBio Laboratories, Carlsbad, CA, United States) following the manufacturer’s instructions. Bacterial RNA was extracted from bulk soil samples as described by Lueders et al. (2004) with appropriate modifications developed for forest soils within the Biodiversity Exploratories (Wüst et al., 2016). Samples were thawed on ice and transferred to a 2-ml screw cap tube containing 0.7 g of sterilized zirconium/silica beads (diameter, 0.1 mm), 750 μl sodium phosphate solution (112.9 mM Na_2_HPO_4_, 7.1 mM NaH_2_PO_4_), and 250 μl TNS-Buffer (500 mM Tris-HCl pH 8, 100 mM NaCl, 10% sodium dodecyl sulfate). Cells were disrupted by bead-beating (2 times at 6.5 m•s^–1^ for 45 s). After centrifugation, samples were extracted with phenol–chloroform–isoamyl alcohol (25:24:1 v/v/v) then chloroform–isoamyl alcohol (24:1), and nucleic acids were pelleted by the addition of polyethylene glycol and centrifugation. Pellets were washed with cold ethanol (70%) and resuspended in 20 to 50 μl Tris-HCl buffer (pH 8.5). RNA was prepared by digestion of co-extracted DNA with RNase free DNase I (ThermoScientific, Waltham, MA, United States) according to the instructions of the manufacturer and subsequently precipitated in sodium acetate (3M, pH 5.2) and isopropanol (99.5%), washed with ethanol (70%, v/v), and resuspended in RNase-free water. Concentrations of RNA were determined using the Quant-iT RiboGreen RNA Assay Kit (Life Technologies, Darmstadt, Germany) and a microtiter plate reader (Tecan Infinite 200 PRO; Männedorf, Switzerland). RNA extracts were treated with a RiboLock RNase inhibitor (final concentration 1 U μl^–1^; Fermentas, Waltham, MA, United States) prior to reverse-transcription PCR. For the synthesis of cDNA from extracted RNA, the GoScript Reverse Transcription System was employed according to the protocol of the manufacturer (Promega, Madison, WI, United States) using random hexamers.

For all samples, biocrusts, and bulk soil, the V3 region of the 16S rRNA was amplified using modified primer pairs 341F (5′-CCTACGGGWGGCWGCAG-3′) and 518R (5′-CCGCGGCTGCTGGCAC-3′) (Muyzer et al., 1993), which contained Illumina adapter sequences and binding sites for sequencing primers. Additionally, the reverse primer included an index region of six nucleotides (Bartram et al., 2011). All samples were amplified in triplicates. The reaction mix (final volume of 50 μl) contained 10 μl PCR buffer (5x; GC Phusion buffer), 1 μl dNTP mix (10 mM each), 0.2 μl each of forward and reverse primers (50 μM each), 1.5 μl dimethyl sulfoxide (DMSO; 100% v/v), and 1 μl Phusion High-Fidelity DNA Polymerase (2 U μl^–1^; Thermo Scientific, Waltham, United States). Amplification proceeded by an initial denaturation step at 94°C for 5 min, followed by 20 cycles at 94°C for 15 seconds, 59°C for 15 seconds, 72°C for 15 seconds, and final extension step at 72°C for 7 minutes. Amplifications were carried out in a Veriti 96-well thermal cycler (Applied Biosystems, Foster city, CA, United States). Amplicons were purified in 2% Metaphor (Lonza group, Basel, Switzerland) agarose gels to allow the separation of products from primers and primer dimers. Subsequently, the PCR products were cleaned with a NucleoSpin Gel and PCR Clean-up Kit (Macherey-Nagel, Düren, Germany) and quantified using a Qubit^®^ dsDNA HS Assay Kit (Life Technologies, Carlsbad, CA, US). Quality was checked with a 2100 Bioanalyzer (Agilent Technologies, Santa Clara, CA, United States), and triplicates from amplification step were pooled in equal amounts. Sequencing was performed on a HiSeq 2500 (Illumina, San Diego, CA, United States) in a paired-end run, yielding a total of 4.70 × 10^8^ sequence reads of 100-bp length. The raw sequencing data were deposited in the European Nucleotide Archive (ENA) under the project PRJEB50153.

Raw sequence reads were processed using plugins available in the QIIME 2™ platform (Version 2017.12)^1^ (Caporaso et al., 2010). Raw sequence reads were imported, joined, quality filtered, chimera checked, and denoised, and the distinct sequences obtained were subsequently allocated to samples using the plugins “vsearch,” “quality-filter,” and “deblur” (Bokulich et al., 2013; Amir et al., 2017). In detail, the QIIME2 default parameters for quality filtering and chimera checking and removal were used. These are listed in the qiime2 command line interface scripts at https://docs.qiime2.org/2017.12/plugins/available/quality-filter/q-score-joined/ and at https://docs.qiime2.org/2017.12/plugins/available/deblur/denoise-16S/.

First, the reads were trimmed in deblur at a length of 165 bases. Then, briefly, read check and chimera check were performed in the plugin “deblur” as a twofold step. The first step involves a “positive filtering by comparison to a database of known sequences (by default the Greengenes 13_8 88% OTUs).” The second step addresses “chimeras originating from PCR amplification prior to sequencing. Therefore, the reads are additionally filtered for *de novo* chimeras using VSEARCH with non-default parameters: –dn = 0.000001, –xn = 1000, –minh = 10000000, –mindiffs = 5.” The above details are quotes from the Supplementary Text 1 of Amir et al. (2017), which contains further details and references with respect to the chimera check procedure.

The representative sequences were aligned to reconstruct a midpoint-rooted tree using the plugins “feature-table,” “alignment,” and “phylogeny.” The taxonomic classification of representative sequences was performed using a Naïve Bayes classifier, which was trained using the 16S rRNA gene sequence database of SILVA (version 132) using the plugin “feature-classifier.” For further statistical analysis, the read abundance table, the taxonomic classification, and the phylogenetic tree were imported into the R package “phyloseq” version 1.24 (McMurdie and Holmes, 2013).

### Statistical Analysis

All statistical analyses were conducted in R Version 4.0.2 for Windows (R Development Core Team, 2009). For correlation analyses of alpha diversity (i.e., richness or number of OTUs) with environmental factors including the sampling site, ANOVA was conducted; the best predictors for the alpha diversity variance were selected by backward elimination stepwise regression analysis based on the AIC (Akaike information criterion) using the “step” command in R (stats package). Environmental data were standardized to account for different scales before analysis. For categorical data, *post hoc* Tukey test was performed after ANOVA. Furthermore, the alpha diversity and significant environmental data were included in a generalized linear model using the “glm” command. For correlation analyses of beta diversity, PERMANOVA with the command “Adonis” from the vegan package (Anderson, 2001) was applied using the Bray–Curtis dissimilarity index, including a permutation test with 1,000 permutations. Ancom analyses was used to identify bacteria families that were significantly more or less abundant in biocrust samples compared to bulk soil (Mandal et al., 2015).

## Results

### Biocrust Occurrence and Elemental Contents of Biocrusts

**FIGURE 1:**
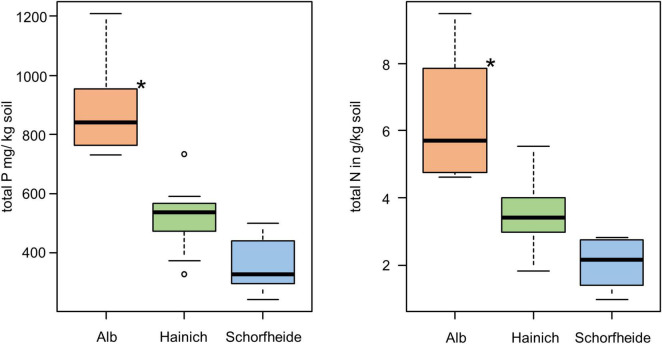
Boxplot of total P content **(left)** and total N **(right)** in biocrusts of three sampling sites; significant differences are indicated by asterisks (ANOVA, Tukey *post hoc*); *n* = 9 for each sampling site.

We analyzed the bacterial community of 27 biocrust samples from all three sampling sites (Alb, Hainich, and Schorfheide) and measured the element content of these biocrusts. Particularly, the total C, N, S, and P content was measured, and the C/N ratio was calculated (Table 1). Phosphorus, N, and C content differed between the three sampling sites: in the Alb samples, the P, N, and C content was significantly higher than in Hainich, and Schorfheide biocrusts had the lowest contents (Figure 1).

**TABLE 1:** Concentration of total carbon, nitrogen, sulfur, and phosphorus in biocrusts from three sampling sites in Germany: A-Alb, H-Hainich, and S-Schorfheide.

Plot	C in g/kg	N in g/kg	S in g/kg	C/N Ratio	P in mg/kg
A1	61.6	4.7	0.5	13.2	730.4
A2	167	9.5	0.9	17.6	954.5
A3	70.3	4.8	0.6	14.8	762.4
A8	68.2	5.2	0.7	13.3	841.3
A9	57.9	4.6	0.6	12.6	815.9
A22	89.3	6.1	0.8	14.6	755.2
A33	120.9	8.2	1	14.8	1209.3
A34	142.4	7.8	0.8	18.1	1093.4
A42	69.6	5.7	0.7	12.2	949.4
H2	24	2	0.6	11.7	590.9
H3	76.1	4.5	0.6	16.9	498.6
H7	38.7	3	0.4	12.9	555.3
H8	52.1	4	0.6	13.0	473.1
H9	37.2	3.1	0.4	12.1	326.5
H10	25.8	1.9	0.3	13.9	536.0
H11	43.2	3.4	0.4	12.6	733.1
H12	53.8	3.5	0.4	15.3	372.0
H13	96.9	5.5	0.7	17.5	567.0
S1	58	2.8	0.9	20.5	340.2
S6.1	38	2.7	0.6	13.8	445.2
S7	11.8	1	0.2	11.9	309.6
S8	36.5	2.5	0.3	14.9	282.6
S9	30.4	1.9	0.3	16.1	314.1
S40	17.1	1.2	0.2	13.8	500.4
S43	48.5	2.8	0.3	17.6	241.1

### Algal Species in Biocrusts

**FIGURE 2:**
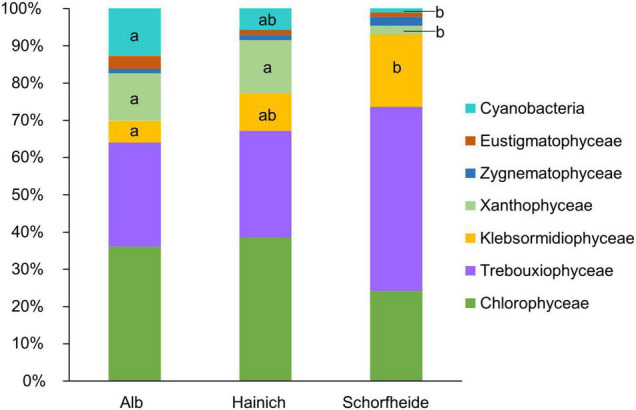
Taxonomic composition of algae community on class level in biocrusts from temperate forests at three sampling sites; significant differences between the three sites were estimated by ANOVA followed by *post hoc* Tukey test and are indicated with letters; *n* = 9 for each sampling site.

In 27 biocrusts from three sampling sites, we identified in total 71 algae and cyanobacteria morphotypes in the cultivated enrichments. In median, we observed 8 different algae species based on morphological identification, with at least 3 and maximum 11 species per biocrust sample. At all three sampling sites, Alb, Hainich, and Schorfheide, Chlorophyta (comprising *Chlorophyceae* and *Trebuxiophyceae*) was the dominant phylum (Figure 2). The three sites, especially Schorfheide, differed remarkably in the relative composition of algae: particularly the richness of cyanobacteria, Stramenopiles (primarily *Xanthophyceae*), and Streptophyta (only *Klebsormidiophyceae*) was significantly different between the three sampling sites. Most cyanobacteria were found in Alb, less in Hainich, and only one Cyanobacterium in Schorfheide. More *Xanthophyceae* were observed in Hainich and Alb than in Schorfheide. Klebsormidiophyceae, in contrast, were more frequently found in Schorfheide than at the other sites. In each biocrust, at least one filamentous alga (like species from the genera *Klebsormidium*, *Xanthonema*, *Microcoleus*, or *Nostoc*) was detected.

### Bacterial Alpha Diversity

High-throughput-sequencing allows a deep insight into the bacterial community because of the high number of sequence reads, irrespective of DNA- and RNA-based analyses. In our study, the number of reads was much higher in the bulk soil sampling batch (RNA) than in the biocrust samples (DNA). After rarefaction, nearly all biocrust samples still reached the saturation in rarefaction analyses, but some bulk soil samples did not reach the saturation in rarefaction analyses. Thus, we might underestimate the diversity of bacteria in bulk soil samples. Nevertheless, we are convinced that our approach was suitable for this kind of analysis because we rather focused on the bacteria communities in biocrust than to describe the bacteria community in bulk soil in detail.

The most abundant bacteria phyla in the biocrust and the bulk soil were the Proteobacteria, followed by Actino- and Acidobacteria (Supplementary Table 1). Proteobacteria alone accounted for nearly half of the biocrust bacteria OTUs (44.7%).

**FIGURE 3:**
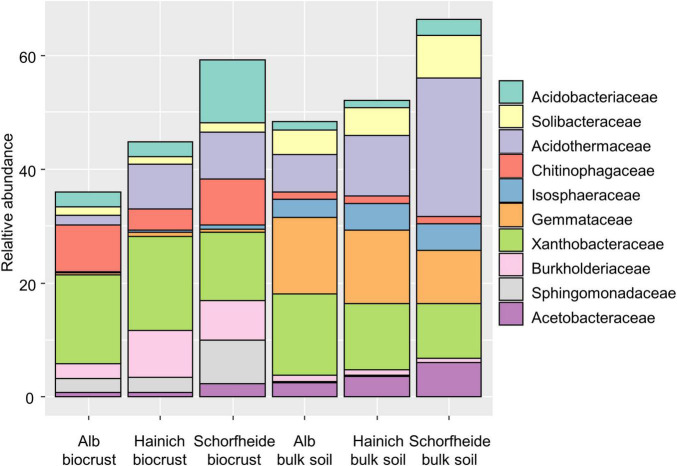
Taxonomic composition of bacteria on the family level in biocrusts and bulk soil from three sampling sites. Families with the highest relative abundance (>5%) are shown; *n* = 9 for each sampling site and soil type. Taxonomic composition on the phylum level is given in Supplementary Table 2.

The ten most abundant bacterial families (relative abundance in either biocrusts or bulk soil > 5%) differed in their relative abundance in the biocrust compared to bulk soil (Figure 3 and Supplementary Table 2). In particular, *Chitinophagaceae, Sphingomonadaceae*, and *Burkolderiaceae* were more abundant in the biocrust. Vice versa, *Isosphaeraceae, Solibacteraceae*, and *Gemmataceae* were more abundant in the bulk soil. Other families did not differ in their relative abundance between the two habitats like *Acidobacteriaceae* and *Xanthobacteraceae*.

Additionally, we performed Ancom analyses in order to identify bacterial families that were significantly enhanced in biocrust or in bulk soil, respectively (Supplementary Table 2). For example, we found significantly more *Bdellovibrionaceae* in biocrust than in bulk soil samples. Members of this group are known bacterivores. Also, we found significantly more *Chitinophagaceae*, *Cytophagaceae*, and *Streptomycetaceae* in biocrusts. All three bacteria families comprise many members that could hydrolyze chitin, cellulose or lignin, respectively. Also, two families with high relevance for the nitrogen cycle were significantly more abundant in biocrusts: *Nitrosomonadaceae* are nitrifying bacteria and some *Pseudomonas* (the only detected genus in the *Pseudomonadaceae*) are denitrifier. *Rhodobacteraceae* were also more abundant in biocrust; they inherit denitrifiers, too. Furthermore, *Rhodobacter* as a photosynthetic member of this family was detected only in biocrusts.

### Environmental Factors Driving Algal and Bacterial Diversity

**FIGURE 4:**
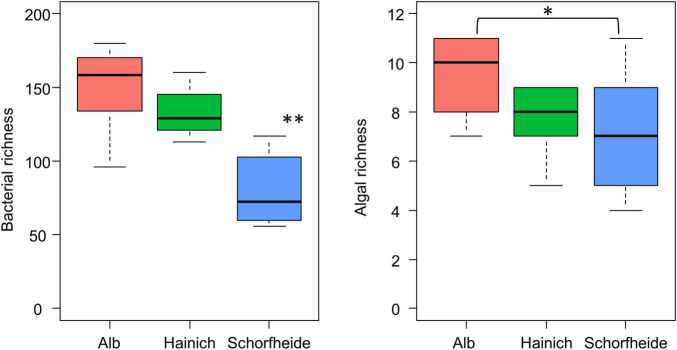
Boxplot visualizes the number of algal species **(right)** and of bacterial OTUs **(left)** in biocrusts from three sites (*n* = 9 per site); asterisk indicates significant differences (based on ANOVA followed by Tukey *post hoc* test): *p < 0.05, ^**^p < 0.01.

We observed much lower bacteria OTU richness in Schorfheide than in Alb or Hainich. The number of identified algal species was also lower in Schorfheide (Figure 4). However, the richness of bacterial OTUs was not correlated with the richness of algal species.

**FIGURE 5:**
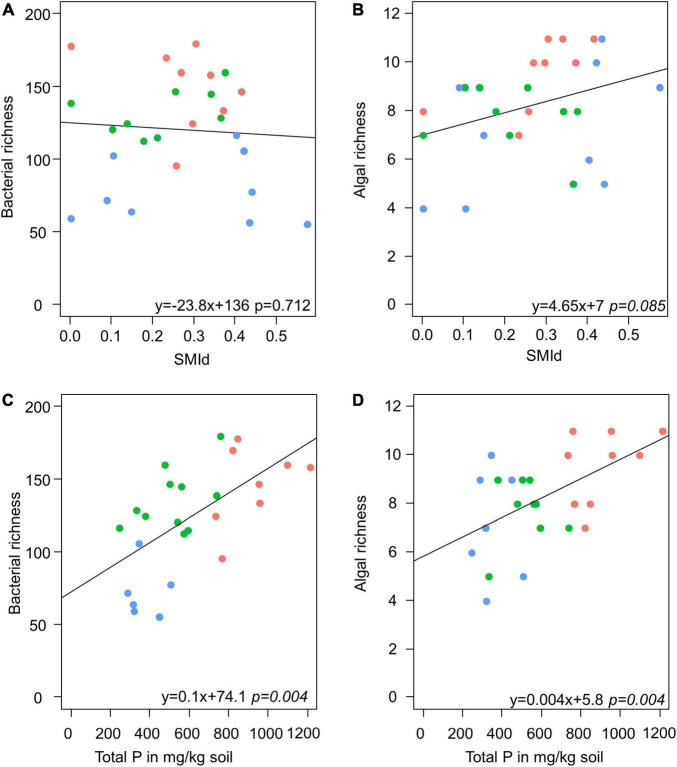
Scatter plots visualize the correlation of stand density (as SMId—see Materials and Methods) with richness of bacteria **(A)** and alga **(B)** and the correlation of total P with richness of bacteria **(C)** and alga **(D)** in biocrusts. Red: Alb; green: Hainich; blue: Schorfheide. The results of a linear model are given below.

Model selection based on the lowest AIC identified the similar environmental factors as a major driver for bacterial as for algae species richness besides the sampling site: stand density (SMId) and total P (Table 2). The SMId was significantly correlated with the algal richness (Figures 5A,B), ranging from natural forest (0) to beech forest thickened with shelter wood (0.57). Nevertheless, we observed that only in Schorfheide the algal richness was correlated with the SMId; in Alb and Hainich, algal richness was independent of SMId. The bacterial OTU richness and the algal species richness were correlated with the P content across the three sampling sites (Figures 5C,D).

**TABLE 2:** Correlation of environmental factors with the richness (based on ANOVA results) and the community composition (based on PERMANOVA results) of algae and bacteria; Signif. codes: ***p < 0.001, **p < 0.01, *p < 0.05,° p < 0.1; n.s.—not significant.

	Richness		Beta diversity	
	Algae	Bacteria	Algae	Bacteria
Sampling site	n.s.	***	***	***
SMId	**	°	n.s.	n.s.
pH	n.s.	°	n.s.	**
N	n.s.	n.s.	*	*
P	n.s.	n.s.	n.s.	n.s.

PERMANOVA was conducted to identify environmental parameters that correlate with the community composition. Also, on the level of beta-diversity, similar environmental drivers were identified that shaped the community composition of algae and bacteria (Table 2). The most important driver was again the sampling site, with Schorfheide as the most different site compared to Alb and Hainich. For algal as well as bacterial community composition, the content of N and P in the biocrusts was important. Total P differed between the sampling sites and thus was not significant in the co-analysis with the sampling site (Figure 1). This means we cannot conclude from our data if the content of P influences the community because the effects might be masked due to the overall differences of the sites.

### Impact of Functional Algal Groups on Bacteria

**FIGURE 6:**
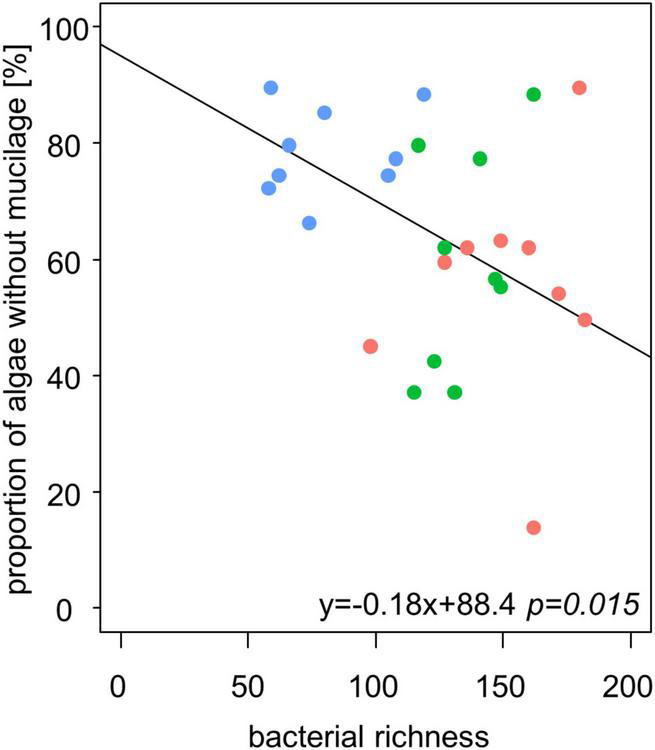
The bacterial richness correlates negatively with the relative content of alga species without mucilage in biocrusts from temperate forests (*n* = 27). Line represents the result of a linear model.

We observed higher bacterial richness when the proportion of algae with mucilage on total algal species was higher (Table 3 and Figure 6). It is important to note that also the proportion of algae with mucilage depends on the sampling site. In Schorfheide, we observed a lower proportion of algal species with mucilage than in the other sites (p < 0.01, ANOVA, Tukey *post hoc*).

**TABLE 3:** Correlation of bacterial richness with functional groups of algae selected according to the results of model selection by AIC; Signif. codes: **p < 0.01, *p < 0.05.

	Df	Sum squares	*F*-value	*P*-value	
No mucilage	1	5,934,091	13.352	0.00114	**
Nanosize	1	14,380	0.032	0.85864	
Microsize	1	2,807,452	6.317	0.01849	*
Residuals	26	11,554,980			

## Discussion

### General Description of Eukaryotic Algae

We observed eight algae per biocrust (median) with a maximum of 11 species. The number of algae species in our study is lower compared to previous studies on biocrusts from Germany with up to 40 species per sample on sandy soils (Langhans et al., 2009; Schulz et al., 2016). Nevertheless, the species richness in the biocrusts from forest in Hainich and Alb was in the similar range compared with that in biocrusts from forest in Schorfheide, which was published earlier (Glaser et al., 2018).

The most abundant algal phylum was Chlorophyta. This observation is typical for terrestrial habitats in temperate regions (Büdel et al., 2016). Interestingly, we observed a gradient from Alb over Hainich to Schorfheide in terms of relative abundance of cyanobacteria, which decreased, and Streptophyta, which increased. The low relative abundance of cyanobacteria in Schorfheide can be explained by the lower pH of around 3.5 in that region (Kaiser et al., 2016) compared to Hainich and Alb (pH ∼ 5) because cyanobacteria are typically rare in acidic forest soils (Hoffmann et al., 2007). In Schorfheide, we frequently observed the algal genus *Klebsormidium* (Streptophyta), but genus *Xanthonema* (Ochrophyta) was rare. In Alb and Hainich, the opposite was found: few *Klebsormidium* species and frequently *Xanthonema* were observed. Both genera are characterized by long, stable filaments, which can potentially initiate biocrust formation. It has been reported that *Klebsormidium* seems to be favored acidic sandy soils, whereas *Xanthonema* is rare in acidic soils (Lukešová, 2001). This corresponds well with Schorfheide pH, which was lower than that at the other two sites (Kaiser et al., 2016), which could explain the more frequent occurrence of *Klebsormidium* at this site.

Filamentous algae or cyanobacteria initiate soil stabilization and biocrust formation. These organisms interweave with the soil matrix and stick the particles together by means of large amounts of polymeric substances (EPS) (Belnap and Büdel, 2016). Thus, it does not surprise that every biocrust in this study contained at least one filamentous algal or cyanobacterial species. This points to the high importance of filamentous species for biocrust formation even in well-developed soils such as European forest soils. This is in congruence with observation from (semi)arid sites and coastal sand dunes, where also filamentous species were abundant in the early stage of biocrusts (Schulz et al., 2016; Weber et al., 2016).

### Biocrust Bacterial Communities

The general composition of phyla in the bulk soil samples was equal to those described three years earlier for these sites (Kaiser et al., 2016), indicating a stable bacterial community in forest soils irrespective of the nucleic acids analyzed. The bulk soil data of this study rely on RNA, while Kaiser et al. (2016) used DNA, which indicates the compatibility of these approaches at the investigated areas. In contrast to bulk soil and the biocrust algal and cyanobacterial composition, bacterial communities in biocrusts from temperate forests cannot be compared with literature since there are no data available so far. In dry land studies, a much smaller proportion of Proteobacteria and higher proportion of cyanobacteria than in our study was found (Kuske et al., 2012; Steven et al., 2013; Maier et al., 2018). The latter is obvious because cyanobacteria dominate biocrusts in arid regions due to their pronounced desiccation tolerance. Bacterial communities of cold region biocrusts are dominated by cyanobacteria and Proteobacteria, even though eukaryotic algae were present and abundant (Rippin et al., 2018b).

Specifics of biocrust bacteria communities can be addressed in our study by comparison to bulk soil samples. We observed higher richness in bulk soil than in the biocrust. This observation is in congruence with previous studies from sandy soils in (semi)-arid regions (Abed et al., 2019; Moreira-Grez et al., 2019; Pombubpa et al., 2020), where also higher bacterial was observed in the biocrust compared to bare soil. In contrary, studies from loess plateau in humid regions and from weakly developed soils in arid regions revealed higher bacterial richness in biocrust than in the bare soil (Xiao and Veste, 2017; Chilton et al., 2018; Maier et al., 2018). In the latter studies, the authors reasoned that the lower diversity was caused by the low organic content and high mobility of the sand in bulk soil. It is clearly a completely different situation in forest ecosystems: here, fully developed, organic-rich, and stable soils dominate, in which a rich bacterial community can develop and sustain even without the support of the biocrust phototrophic communities.

We hypothesized that the bacteria in biocrusts are a subset of the overall soil bacteria. Indeed, a closer look revealed that the bacterial communities of biocrusts and bulk soil differed strongly. This means that the bacterial biocrust community seems to be only a subset of the soil bacterial community. The differences may result from the biocrust phototrophic community, which support a unique bacterial community composition. A deeper look into the respective OTUs should clarify which functional traits of bacteria might be favored in biocrusts.

Significantly more *Nitrosomonadaceae* and *Pseudomonadaceae* (*Pseudomonas* sp.) OTUs were observed in biocrust than in bulk soil samples. Many members of this group are nitrifying and denitrifying bacteria. Their higher relative abundance might point to a more active nitrogen cycle in biocrust compared to bulk soil. A previous study at the same sites observed no clear pattern comparing gene abundance assigned to N-mineralization when comparing biocrust and bare soil (Kurth et al., 2020). This finding further supports the idea of higher nitrogen turnover N in biocrusts compared to bulk soil. Previous studies showed a high N-fixation potential in biocrusts compared to trampled soil (Steven and Kuske, 2018). In our study sites, we observed nitrogen-fixing cyanobacteria in low abundances with one OTU assigned to *Nostoc*. Also non-photosynthetic diazotrophic genera known from early-stage biocrusts like *Shigella*, *Klebsiella*, and *Ideonella* were missing in our study (Pepe-Ranney et al., 2016). This points toward no N-limitation and an efficient N-recycling in well-developed forest soils in temperate regions.

Also, *Chitinophagaceae*, *Cytophagaceae*, and *Streptomycetaceae* appeared more often in biocrusts than in bulk soils. Members of these bacterial families are able to hydrolyze chitin, cellulose, or lignin, respectively. A higher relative proportion of these families point toward a fast recycling of C-compounds and a higher content of such complex C-compounds in the organic matter in biocrusts. Also a previous study reported more Chitinophagaceae and Cytophagaceae in biocrust compared to bulk soil (Maier et al., 2018). Furthermore, this observation is in congruence with a study on chitinase activity in biocrusts, which also observed a much higher activity in biocrusts compared to biocrust-free soil (Brankatschk et al., 2013). In biocrusts from forest, significantly more fungi and microfauna were reported compared to bulk soil (Ngosong et al., 2020). The cell walls of microalgae and mosses contain high proportions of cellulose. A higher density of cell-wall-containing organisms could explain also the higher relative abundance of cellulose-hydrolyzing bacteria in biocrusts.

A significantly higher relative abundance of *Bdellovibrionaceae* was observed in biocrusts than in bulk soil samples. This bacterial family is very interesting because it inherits bacterivores. Indeed, the genus *Bdellovibrio* was frequently detected in biocrusts (in total 107 different OTUs). The interesting fact about bacterivore bacteria is that they diverge from the classical position of bacteria in the microbial loop. In contrast to phages, *Bdellovibrio* has a broader host range and feeds on different bacterial species and genera. Although it is known to be widely distributed, its impact on the bacterial community is not well-defined (Johnke et al., 2014). A higher relative abundance of the family members might be due to an increased bacterial density in biocrusts. Although bacterial biomass was not measured in our study, other studies already confirmed a higher bacterial biomass (based on lipid analyses) in biocrusts compared to bulk soils (Ngosong et al., 2020). Another study from the same forest sites of all three regions found only a slightly higher bacterial abundance (analyzed by quantitative PCR) in biocrust compared to biocrust-free top soil stating that rich forest soils already allow high microbial abundances at the surface (Kurth et al., 2020). Thus, we assume that biocrusts in the forest generally have a higher bacterial density compared to deeper bulk soil where effects of litter are less prominent. The reduction of bacterial biomass in the bulk soil of arid regions is much stronger and has been shown to be as high as 99% compared to the biocrusts while the diversity remained similar (Steven et al., 2013). In our opinion, our results point toward an important functional role of *Bdellovibrio* in terms of nutrient recycling in a microbial hotspot like biocrusts. In previous studies, Bdellovibrio was not mentioned explicitly. However, the Supplementary figure in one study of biocrusts seems not to indicate a significant difference in the relative abundance of Bdellovibrionaceae in biocrusts and bare soil (Maier et al., 2018).

### Bacteria and Algae Interactions

We hypothesized that the algal and bacterial communities are linked to each other. First attempts were already conducted to correlate photosynthetic organisms with the bacterial community by comparing the bacterial community of different biocrust types, which were defined as cyanobacteria, chlorolichen, and moss-dominated (Maier et al., 2018). In our study, the comparison was made on the level of individual algal species with respect to their characteristic features, i.e., potential functional traits in biocrusts.

At first sight, we did not observe a correlation of algae richness with bacterial richness. This means that more different algae species do not necessarily mean higher bacterial OTU richness. This might be due to the organic-rich forest soils, where bacterial community might be less dependent on organic input by microalgae compared to semiarid regions. Nevertheless, similar environmental factors were identified that shape the bacterial and algal communities in biocrusts. The sampling site was the most important factor for the algal and bacterial biocrust communities. In detail, one sampling site (Schorfheide) differed from the other two sites, Alb and Hainich. The geographical distances between the sites are about the same from Schorfheide to Hainich and Hainich to Alb. Hence, biogeography can be excluded as potential reason. The soil in Schorfheide is much sandier, and the pH is lower than in Alb and Hainich (Kaiser et al., 2016). These differences in soil parameters might explain the variation of bacterial community between the sampling sites. Indeed, previous work at the same sampling sites identified also the pH as a major driver of the bacterial community composition, whereas the silvicultural management and the dominant tree species were of minor importance (Kaiser et al., 2016).

The SMId, a measurement for stand density in the context of silvicultural management (Schall and Ammer, 2013), affected the richness of both algae and bacteria in Schorfheide. Stand density influences the light intensity at the forest floor. Especially in spring, the soil surface temperature strongly depends on sunlight hitting the ground, which is crucial for organism activity. Furthermore, higher stand densities lead to higher litter fall and, thus, higher nutrient input to the topsoil layer. The tree species influence the soil pH, e.g., coniferous litter is more acidic than deciduous species, with a higher stand density enhancing this effect (Kaiser et al., 2016). Leaf covers also change the water regime by retention of precipitation and lower the evaporation by shading. Biocrust development is driven by the phototrophic community and consequently depends on their photosynthetic activity. Although the biocrust community in the two regions (Alb and Hainich) was not correlated with the silvicultural management, the overall biocrust occurrence might be influenced by the management. For example, skid trails and clear-cuts disturb the vegetation cover and bare soil gets exposed. At such places, biocrusts can develop fast, whereas in a natural forest, only small-scale disturbances occur, like windfall or digging of holes by wild boars. Thus, silvicultural management might have an impact on the frequency of biocrust occurrence but less on the community composition.

The content of N was correlated with both bacterial and algal community composition. N poor spots promote a microbial community that favored specialists for N-fixation. Nitrification and denitrification are processes mainly driven by microbes. Denitrification would cause a certain N loss from the biocrust system, whereas the transformation of N-containing compounds by nitrifiers stimulates the whole community and might enhance nutrient recycling. Previous studies on biocrusts at different developmental stages observed a higher potential enzyme activity and higher gene abundance in terms of nitrification and denitrification in biocrusts increasing with biocrust development (Brankatschk et al., 2013; Li et al., 2020). In terms of N-fixation, the results were contrasting: one study found higher enzyme activity and copy numbers in biocrust increasing with developmental stage (Brankatschk et al., 2013), whereas another study found lower copy numbers of N-fixation genes in bacterial biocrusts compared to moss biocrusts (Li et al., 2020). This supports the idea that phototrophic community, which changes during biocrust development, is linked to the mainly bacteria-driven nitrogen cycling.

The P content of the biocrust was highly correlated with the bacterial richness. In contrast to N, P cannot be fixed from the atmosphere, which makes it a hard-to-overcome limiting factor. Atmospheric deposition of P by dust or rain is limited (Berthold et al., 2019), and its weathering from minerals is energy-consuming as many strategies require a sufficient C supply. The small inputs of atmospheric P may lead to an accumulation over time in the biocrusts because organic bound P as well as the available P pools undergo rapid transformation by the biocrust biota (Baumann et al., 2017). Thus, recycling of P is the main source for microbial growth and is pronounced in P depleted soils (Jones and Oburger, 2011; Kurth et al., 2020). The size of the P pools consequently shaped the bacterial community and, as with N, favored specialists (Kurth et al., 2020).

The soil pH was correlated with the bacterial community of the bulk soil. Soil pH was often reported as the main driver for bacterial diversity (Lauber et al., 2009). Furthermore, it has been described that the pH is an important factor for algal group dominance, e.g., in acidic soils, mostly green algae are found (Hoffmann, 1989), although reports on a direct link between algae diversity and pH are missing. This may be because algae shape the pH in their closest environment by photosynthetic activity. A study at the same forest sites showed an increase in pH in biocrusts compared to bulk soil for acidic soils, whereas the pH was lowered in already alkaline environments (Kurth et al., 2020).

We observed a positive correlation of mucilage producing algal species and bacterial OTUs in biocrusts. Mucilage sheaths protect algae from desiccation (Holzinger and Karsten, 2013) and coccal algae from grazing by, e.g., amoeba. Although many bacteria species are able to produce EPS, they might grow and use the mucilage sheaths from algae possibly also as a carbon source. It seems reasonable that other organisms use the sheaths of algae and live attached or close by: the production of EPS is “expensive” as a lot of carbon is needed to build this protecting sheath. The positive effect of foreign EPS has been proven in an experiment by Knowles and Castenholz (2008) who could already show that algae without mucilage could cope better with desiccation stress if supported by EPS from other organisms (Knowles and Castenholz, 2008). However, for solid conclusions on commensal live styles, *in vivo* observations would be necessary.

## Conclusion

This study is the first to report on bacteria in biocrust from temperate forests. Although the soil was well developed in the study areas, the bacterial biocrust community differed significantly from the bulk soil bacteria. It seemed that the bacteria were recruited from bulk soil, but due to the activity of phototrophs, excretion of EPS, and generally higher content of nutrients, a different bacterial community could establish. We identified the sampling site, N and P concentration, and the presence of mucilage-excreting algae as important drivers for bacterial biocrust community pattern. In conclusion, biocrusts in European forest seem to be biodiversity hotspots and inherit a specialized bacterial community compared to bulk soil.

## Data Availability Statement

The datasets presented in this study can be found in online repositories. The names of the repository/repositories and accession number(s) can be found in the article/Supplementary Material.

## Author Contributions

KG, KB, and JO developed the idea. KG, KB, and JS collected and analyzed the data. KG wrote the first draft of manuscript which was edited by MA, KB, JO, and JS. All authors edited and approved the final version of this manuscript.

## Conflict of Interest

The authors declare that the research was conducted in the absence of any commercial or financial relationships that could be construed as a potential conflict of interest.

## Publisher’s Note

All claims expressed in this article are solely those of the authors and do not necessarily represent those of their affiliated organizations, or those of the publisher, the editors and the reviewers. Any product that may be evaluated in this article, or claim that may be made by its manufacturer, is not guaranteed or endorsed by the publisher.

## Footnotes

1
https://qiime2.org/

## References

[B1] Abed R. M. M. Tamm A. Hassenrück C. Al-Rawahi A. N. Rodríguez-Caballero E. Fiedler S. (2019). Habitat-dependent composition of bacterial and fungal communities in biological soil crusts from Oman. *Sci. Rep.* 9:6468. 10.1038/s41598-019-42911-6 31015576 PMC6478931

[B2] Albright M. B. N. Mueller R. C. Gallegos-Graves L. V. Belnap J. Reed S. C. Kuske C. R. (2019). Interactions of Microhabitat and Time Control Grassland Bacterial and Fungal Composition. *Front. Ecol. Evol.* 7:367. 10.3389/fevo.2019.00367

[B3] Amir A. McDonald D. Navas-Molina J. A. Kopylova E. Morton J. T. Zech (2017). Deblur Rapidly Resolves Single-Nucleotide Community Sequence Patterns. *mSystems* 0019 e191–e116. 10.1128/mSystems.00191-16 28289731 PMC5340863

[B4] Anderson M. J. (2001). A new method for non-parametric multivariate analysis of variance. *Austral Ecol.* 26 32–46. 10.1111/j.1442-9993.2001.01070.pp.x

[B5] Bartram A. K. Lynch M. D. J. Stearns J. C. Moreno-Hagelsieb G. Neufeld J. D. (2011). Generation of multimillion-sequence 16S rRNA gene libraries from complex microbial communities by assembling paired-end Illumina reads. *Appl. Environ. Microbiol.* 77 3846–3852. 10.1128/AEM.02772-10 21460107 PMC3127616

[B6] Baumann K. Glaser K. Mutz J.-E. Karsten U. MacLennan A. Hu Y. (2017). Biological soil crusts of temperate forests: Their role in P cycling. *Soil Biol. Biochem.* 109 156–166. 10.1016/j.soilbio.2017.02.011

[B7] Belnap J. Büdel B. (2016). “Biological Soil Crusts as Soil Stabilizers,” in *Biological Soil Crusts: an Organizing Principle in Drylands*, eds Weber B. Büdel B. Belnap J. (Cham: Springer International Publishing), 305–320. 10.1007/978-3-319-30214-0_16

[B8] Belnap J. Büdel B. Lange O. L. (2001). “Biological soil crusts: characteristics and distribution,” in *Biological Soil Crusts: Structure, Function, and Management Ecological Studies*, eds Belnap J. Lange O. L. (Berlin, Heidelberg: Springer-Verlag), 3–30.

[B9] Belnap J. Büdel B. Lange O. L. (2003). “Biological Soil Crusts: Characteristics and Distribution,” in *Biological Soil Crusts: Structure, Function, and Management*, eds Belnap J. Lange O. L. (Berlin: Springer Berlin Heidelberg), 3–30. 10.1007/978-3-642-56475-8_1

[B10] Belnap J. Weber B. Büdel B. (2016). “Biological Soil Crusts as an Organizing Principle in Drylands,” in *Biological Soil Crusts: An Organizing Principle in Drylands*, eds Weber B. Büdel B. Belnap J. (Cham: Springer International Publishing), 3–13. 10.1007/978-3-319-30214-0_1

[B11] Beraldi-Campesi H. Hartnett H. E. Anbar A. Gordon G. W. Garcia-Pichel F. (2009). Effect of biological soil crusts on soil elemental concentrations: implications for biogeochemistry and as traceable biosignatures of ancient life on land. *Geobiology* 7 348–359. 10.1111/j.1472-4669.2009.00204.x 19573165

[B12] Berthold M. Wulff R. Reiff V. Karsten U. Nausch G. Schumann R. (2019). Magnitude and influence of atmospheric phosphorus deposition on the southern Baltic Sea coast over 23 years: implications for coastal waters. *Environ. Sci. Eur.* 31 1–11. 10.1186/s12302-019-0208-y

[B13] Bokulich N. A. Subramanian S. Faith J. J. Gevers D. Gordon J. I. Knight R. (2013). Quality-filtering vastly improves diversity estimates from Illumina amplicon sequencing. *Nat. Methods* 10 57–59. 10.1038/nmeth.2276 23202435 PMC3531572

[B14] Borchhardt N. Baum C. Mikhailyuk T. Karsten U. (2017). Biological Soil Crusts of Arctic Svalbard—Water Availability as Potential Controlling Factor for Microalgal Biodiversity. *Front. Microbiol.* 8:1485. 10.3389/fmicb.2017.01485 28848507 PMC5550688

[B15] Brankatschk R. Fischer T. Veste M. Zeyer J. (2013). Succession of N cycling processes in biological soil crusts on a Central European inland dune. *FEMS Microbiol. Ecol.* 83 149–160. 10.1111/j.1574-6941.2012.01459.x 22816620

[B16] Büdel B. Dulić T. Darienko T. Rybalka N. Friedl T. (2016). “Cyanobacteria and Algae of Biological Soil Crusts,” in *Biological Soil Crusts: an Organizing Principle in Drylands*, eds Weber B. Büdel B. Belnap J. (Cham: Springer International Publishing), 55–80. 10.1007/978-3-319-30214-0_4

[B17] Cania B. Vestergaard G. Kublik S. Köhne J. M. Fischer T. Albert A. (2019). Biological Soil Crusts from Different Soil Substrates Harbor Distinct Bacterial Groups with the Potential to Produce Exopolysaccharides and Lipopolysaccharides. *Microb. Ecol.* 79 326–341. 10.1007/s00248-019-01415-6 31372685

[B18] Caporaso J. G. Kuczynski J. Stombaugh J. Bittinger K. Bushman F. D. Costello E. K. (2010). QIIME allows analysis of high-throughput community sequencing data. *Nat. Methods* 7 335–336. 10.1038/nmeth.f.303 20383131 PMC3156573

[B19] Chilton A. M. Neilan B. A. Eldridge D. J. (2018). Biocrust morphology is linked to marked differences in microbial community composition. *Plant Soil* 429 65–75. 10.1007/s11104-017-3442-3

[B20] Corbin J. D. Thiet R. K. (2020). Temperate biocrusts: mesic counterparts to their better-known dryland cousins. *Front. Ecol. Environ.* 18:fee.2234. 10.1002/fee.2234

[B21] Drahorad S. L. Jehn F. U. Ellerbrock R. H. Siemens J. Felix-Henningsen P. (2020). Soil organic matter content and its aliphatic character define the hydrophobicity of biocrusts in different successional stages. *Ecohydrology* 13:e2232. 10.1002/eco.2232

[B22] Dümig A. Veste M. Hagedorn F. Fischer T. Lange P. Spröte R. (2014). Organic matter from biological soil crusts induces the initial formation of sandy temperate soils. *CATENA* 122 196–208. 10.1016/j.catena.2014.06.011

[B23] Fischer M. Bossdorf O. Gockel S. Hänsel F. Hemp A. Hessenmöller D. (2010). Implementing large-scale and long-term functional biodiversity research: The Biodiversity Exploratories. *Basic Appl. Ecol.* 11 473–485. 10.1016/j.baae.2010.07.009

[B24] Glaser K. Baumann K. Leinweber P. Mikhailyuk T. Karsten U. (2018). Algal richness in BSCs in forests under different management intensity with some implications for P cycling. *Biogeosciences* 15 4181–4192. 10.5194/bg-15-4181-2018

[B25] Glaser K. Donner A. Albrecht M. Mikhailyuk T. Karsten U. (2017). Habitat-specific composition of morphotypes with low genetic diversity in the green algal genus *Klebsormidium* (Streptophyta) isolated from biological soil crusts in Central European grasslands and forests. *Eur. J. Phycol.* 52 188–199. 10.1080/09670262.2016.1235730

[B26] Gray D. W. Lewis L. A. Cardon Z. G. (2007). Photosynthetic recovery following desiccation of desert green algae (Chlorophyta) and their aquatic relatives. *Plant Cell Environ.* 30 1240–1255. 10.1111/j.1365-3040.2007.01704.x 17727415

[B27] Gypser S. Herppich W. B. Fischer T. Lange P. Veste M. (2016a). Photosynthetic characteristics and their spatial variance on biological soil crusts covering initial soils of post-mining sites in Lower Lusatia. *NE Germany*. *Flora Morphol. Dist. Funct. Ecol. Plants* 220 103–116. 10.1016/j.flora.2016.02.012

[B28] Gypser S. Veste M. Fischer T. Lange P. (2016b). Infiltration and water retention of biological soil crusts on reclaimed soils of former open-cast lignite mining sites in Brandenburg, north-east Germany. *J. Hydrol. Hydromechanics* 64 1–11. 10.1515/johh-2016-0009

[B29] Gypser S. Veste M. Fischer T. Lange P. (2015). Formation of soil lichen crusts at reclaimed post-mining sites., Lower Lusatia, North-east Germany. Graphis Scripta 27 3–14.

[B30] Hoffmann L. (1989). Algae of terrestrial habitats. *Bot. Rev* 55 77–105. 10.1007/BF02858529

[B31] Hoffmann L. Ector L. Kostikov I. (2007). Algal Flora from Limed and Unlimed Forest Soils in the Ardenne (Belgium). *Syst. Geogr. Plants* 77 15–90.

[B32] Holzinger A. Karsten U. (2013). Desiccation stress and tolerance in green algae: consequences for ultrastructure, physiological and molecular mechanisms. *Front. Plant Sci.* 4:327. 10.3389/fpls.2013.00327 23986769 PMC3749462

[B33] Johnke J. Cohen Y. de Leeuw M. Kushmaro A. Jurkevitch E. Chatzinotas A. (2014). Multiple micro-predators controlling bacterial communities in the environment. *Curr. Opin. Biotechnol.* 27 185–190. 10.1016/j.copbio.2014.02.003 24598212

[B34] Johnson S. L. Neuer S. Garcia-Pichel F. (2007). Export of nitrogenous compounds due to incomplete cycling within biological soil crusts of arid lands. *Environ. Microbiol.* 9 680–689. 10.1111/j.1462-2920.2006.01187.x 17298368

[B35] Jones D. L. Oburger E. (2011). “Solubilization of Phosphorus by Soil Microorganisms,” in *Phosphorus in Action: Biological Processes in Soil Phosphorus Cycling*, eds Bünemann E. Oberson A. Frossard E. (Berlin: Springer), 169–198. 10.1007/978-3-642-15271-9_7

[B36] Juottonen H. Männistö M. Tiirola M. Kytöviita M. (2020). Cryptogams signify key transitions of bacteria and fungi in Arctic sand dune succession. *New Phytol.* 226 1836–1849. 10.1111/nph.16469 32017117

[B37] Kaiser K. Wemheuer B. Korolkow V. Wemheuer F. Nacke H. Schöning I. (2016). Driving forces of soil bacterial community structure, diversity, and function in temperate grasslands and forests. *Sci. Rep.* 6:33696. 10.1038/srep33696 27650273 PMC5030646

[B38] Karsten U. Lütz C. Holzinger A. (2010). Ecophysiological performance of the aeroterrestrial green alga Klebsormidium crenulatum (Charophyceae, Streptophyta) isolated from an alpine soil crust with emphasis on desiccation stress. *J. Phycol.* 46 1187–1197. 10.1111/j.1529-8817.2010.00921.x 27021989

[B39] Knowles E. J. Castenholz R. W. (2008). Effect of exogenous extracellular polysaccharides on the desiccation and freezing tolerance of rock-inhabiting phototrophic microorganisms: Effect of EPS on tolerance of rock-inhabiting phototrophs. *FEMS Microbiol. Ecol.* 66 261–270. 10.1111/j.1574-6941.2008.00568.x 18710394

[B40] Kurth J. K. Albrecht M. Karsten U. Glaser K. Schloter M. Schulz S. (2020). Correlation of the abundance of bacteria catalyzing phosphorus and nitrogen turnover in biological soil crusts of temperate forests of Germany. *Biol. Fertil. Soils* 14 179–192. 10.1007/s00374-020-01515-3

[B41] Kuske C. R. Yeager C. M. Johnson S. Ticknor L. O. Belnap J. (2012). Response and resilience of soil biocrust bacterial communities to chronic physical disturbance in arid shrublands. *ISME J.* 6 886–897. 10.1038/ismej.2011.153 22113374 PMC3309361

[B42] Langhans T. M. Storm C. Schwabe A. (2009). Community Assembly of Biological Soil Crusts of Different Successional Stages in a Temperate Sand Ecosystem, as Assessed by Direct Determination and Enrichment Techniques. *Microbiol. Ecol.* 58 394–407. 10.1007/s00248-009-9532-x 19479305

[B43] Lauber C. L. Hamady M. Knight R. Fierer N. (2009). Pyrosequencing-Based Assessment of Soil pH as a Predictor of Soil Bacterial Community Structure at the Continental Scale. *Appl. Environ. Microbiol.* 75 5111–5120. 10.1128/AEM.00335-09 19502440 PMC2725504

[B44] Li J.-Y. Jin X.-Y. Zhang X.-C. Chen L. Liu J.-L. Zhang H.-M. (2020). Comparative metagenomics of two distinct biological soil crusts in the Tengger Desert. *China. Soil Biol. Biochem.* 140:107637. 10.1016/j.soilbio.2019.107637

[B45] Lueders T. Manefield M. Friedrich M. W. (2004). Enhanced sensitivity of DNA- and rRNA-based stable isotope probing by fractionation and quantitative analysis of isopycnic centrifugation gradients. *Environ. Microbiol.* 6 73–78. 10.1046/j.1462-2920.2003.00536.x 14686943

[B46] Lukešová A. (2001). Soil algae in brown coal and lignite post-mining areas in Central Europe (Czech Republic and Germany). *Restor. Ecol.* 9 341–350. 10.1046/j.1526-100X.2001.94002.x

[B47] Maier S. Tamm A. Wu D. Caesar J. Grube M. Weber B. (2018). Photoautotrophic organisms control microbial abundance, diversity, and physiology in different types of biological soil crusts. *ISME J.* 12 1032–1046. 10.1038/s41396-018-0062-8 29445133 PMC5864206

[B48] Mandal S. Van Treuren W. White R. A. Eggesbø M. Knight R. Peddada S. D. (2015). Analysis of composition of microbiomes: a novel method for studying microbial composition. *Microbial. Ecol. Health Dis.* 26:27663. 10.3402/mehd.v26.27663 26028277 PMC4450248

[B49] McMurdie P. J. Holmes S. (2013). phyloseq: An R Package for Reproducible Interactive Analysis and Graphics of Microbiome Census Data. *PLoS One* 8:e61217. 10.1371/journal.pone.0061217 23630581 PMC3632530

[B50] Miralles I. Trasar-Cepeda C. Soria R. Ortega R. Lucas-Borja M. E. (2021). Environmental and ecological factors influencing soil functionality of biologically crusted soils by different lichen species in drylands. *Sci. Total Environ.* 794:148491. 10.1016/j.scitotenv.2021.148491 34217081

[B51] Moreira-Grez B. Tam K. Cross A. T. Yong J. W. H. Kumaresan D. Nevill P. (2019). The Bacterial Microbiome Associated With Arid Biocrusts and the Biogeochemical Influence of Biocrusts Upon the Underlying Soil. *Front. Microbiol.* 10:2143. 10.3389/fmicb.2019.02143 31608023 PMC6768011

[B52] Muyzer G. De Waal E. C. Uitterlinden A. G. (1993). Profiling of complex microbial populations by denaturing gradient gel electrophoresis analysis of polymerase chain reaction-amplified genes coding for 16S rRNA. *Appl. Environ. Microbiol.* 59 695–700. 10.1128/aem.59.3.695-700.1993 7683183 PMC202176

[B53] Ngosong C. Buse T. Ewald M. Richter A. Glaser K. Schöning I. (2020). Influence of management intensity and environmental conditions on microbiota in biological soil crust and crust-free soil habitats of temperate forests. *Soil Biol. Biochem.* 144:107761. 10.1016/j.soilbio.2020.107761

[B54] Pepe-Ranney C. Koechli C. Potrafka R. Andam C. Eggleston E. Garcia-Pichel F. (2016). Non-cyanobacterial diazotrophs mediate dinitrogen fixation in biological soil crusts during early crust formation. *ISME J.* 10 287–298. 10.1038/ismej.2015.106 26114889 PMC4737922

[B55] Pombubpa N. Pietrasiak N. De Ley P. Stajich J. E. (2020). Insights into drylands biocrust microbiome: geography, soil depth, and crust type affect biocrust microbial communities and networks in Mojave Desert. *U.S.A. FEMS Microbiol. Ecol.* 96:fiaa125. 10.1093/femsec/fiaa125 32573682 PMC7426032

[B56] Pushkareva E. Johansen J. R. Elster J. (2016). A review of the ecology, ecophysiology and biodiversity of microalgae in Arctic soil crusts. *Polar Biol.* 39 2227–2240. 10.1007/s00300-016-1902-5

[B57] R Development Core Team. (2009). *R: A language and environment for statistical computing.* Vienna, AUS: R Foundation for Statistical Computing.

[B58] Ramanan R. (2016). Algae–bacteria interactions: Evolution, ecology and emerging applications. *Biotechnol. Adv.* 34 14–29. 10.1016/j.biotechadv.2015.12.003 26657897

[B59] Rieser J. Veste M. Thiel M. Schönbrodt-Stitt S. (2021). Coverage and Rainfall Response of Biological Soil Crusts Using Multi-Temporal Sentinel-2 Data in a Central European Temperate Dry Acid Grassland. *Remote Sens.* 13:3093. 10.3390/rs13163093

[B60] Rippin M. Borchhardt N. Williams L. Colesie C. Jung P. Büdel B. (2018a). Genus richness of microalgae and Cyanobacteria in biological soil crusts from Svalbard and Livingston Island: morphological versus molecular approaches. *Polar Biol.* 41 909–923. 10.1007/s00300-018-2252-2

[B61] Rippin M. Lange S. Sausen N. Becker B. (2018b). Biodiversity of biological soil crusts from the Polar Regions revealed by metabarcoding. *FEMS Microbiol. Ecol.* 94:fiy036. 10.1093/femsec/fiy036 29514253

[B62] Samolov E. Baumann K. Büdel B. Jung P. Leinweber P. Mikhailyuk T. (2020). Biodiversity of Algae and Cyanobacteria in Biological Soil Crusts Collected Along a Climatic Gradient in Chile Using an Integrative Approach. *Microorganisms* 8:1047. 10.3390/microorganisms8071047 32674483 PMC7409284

[B63] Schall P. Ammer C. (2013). How to quantify forest management intensity in Central European forests. *Eur. J. Forest Res.* 132 379–396. 10.1007/s10342-013-0681-6

[B64] Schelhaas M.-J. Nabuurs G.-J. Schuck A. (2003). Natural disturbances in the European forests in the 19th and 20th centuries. *Glob. Change Biol.* 9 1620–1633. 10.1046/j.1365-2486.2003.00684.x

[B65] Schulz K. Mikhailyuk T. Dreßler M. Leinweber P. Karsten U. (2016). Biological Soil Crusts from coastal dunes at the Baltic Sea: cyanobacterial and algal biodiversity and related soil properties. *Microbial. Ecol.* 71 178–193. 10.1007/s00248-015-0691-7 26507846

[B66] Seitz S. Nebel M. Goebes P. Käppeler K. Schmidt K. Shi X. (2017). Bryophyte-dominated biological soil crusts mitigate soil erosion in an early successional Chinese subtropical forest. *Biogeosciences* 14 5775–5788. 10.5194/bg-14-5775-2017

[B67] Solly E. F. Schöning I. Boch S. Kandeler E. Marhan S. Michalzik B. (2014). Factors controlling decomposition rates of fine root litter in temperate forests and grasslands. *Plant Soil* 382 203–218. 10.1007/s11104-014-2151-4

[B68] Steven B. Gallegos-Graves L. V. Belnap J. Kuske C. R. (2013). Dryland soil microbial communities display spatial biogeographic patterns associated with soil depth and soil parent material. *FEMS Microbiol. Ecol.* 86 101–113. 10.1111/1574-6941.12143 23621290

[B69] Steven B. Kuske C. (2018). Chronic Physical Disturbance Substantially Alters the Response of Biological Soil Crusts to a Wetting Pulse, as Characterized by Metatranscriptomic Sequencing. *Front Microbiol* 9:2382 10.1101/326181 30349515 PMC6186815

[B70] Szyja M. Büdel B. Colesie C. (2018). Ecophysiological characterization of early successional biological soil crusts in heavily human-impacted areas. *Biogeosciences* 15 1919–1931. 10.5194/bg-15-1919-2018

[B71] Weber B. Bowker M. Zhang Y. Belnap J. (2016). “Natural Recovery of Biological Soil Crusts After Disturbance,” in *Biological Soil Crusts: an Organizing Principle in Drylands*, eds Weber B. Büdel B. Belnap J. (Cham: Springer International Publishing), 479–498. 10.1007/978-3-319-30214-0_23

[B72] Wüst P. K. Nacke H. Kaiser K. Marhan S. Sikorski J. Kandeler E. (2016). Estimates of Soil Bacterial Ribosome Content and Diversity Are Significantly Affected by the Nucleic Acid Extraction Method Employed. *Appl. Environ. Microbiol.* 82 2595–2607. 10.1128/AEM.00019-16 26896137 PMC4836429

[B73] Xiao B. Veste M. (2017). Moss-dominated biocrusts increase soil microbial abundance and community diversity and improve soil fertility in semi-arid climates on the Loess Plateau of China. *Appl. Soil Ecol.* 11 165–177. 10.1016/j.apsoil.2017.05.005

