# Supplementary material for: Biological Soil Crust From Mesic Forests Promote a Specific Bacteria Community
Source: Front Microbiol. 2022 Mar 16;13:769767. doi: 10.3389/fmicb.2022.769767 (PMC8966483; doi:10.3389/fmicb.2022.769767)
Supplement: Supplementary file 1 [file Table_1.DOCX]

Table S1. Overview about characteristics of the sampling locations; these data are public available at the Bexis data bank: latitude and longitude under dataset number 1000; SMI and SMId under number 17746; pH under number 19067; sand, silt and clay content under number 14686

| Plot | Region | Latitude | Longitude | main tree species | SMId | SMI | pH | Clay | silt | sand |
| --- | --- | --- | --- | --- | --- | --- | --- | --- | --- | --- |
| A01 | Alb | 48.48 | 9.33 | Spruce | 0.295 | 0.54 | 3.385 | 318 | 659 | 23 |
| A02 | Alb | 48.38 | 9.35 | Spruce | 0.37 | 0.568 | 4.385 | 500 | 462 | 38 |
| A03 | Alb | 48.41 | 9.36 | Spruce | 0.255 | 0.515 | 5.045 | 527 | 424 | 49 |
| A08 | Alb | 48.38 | 9.38 | Beech | 0 | 0.011 | 6.145 | 492 | 466 | 42 |
| A09 | Alb | 48.37 | 9.42 | Beech | 0.232 | 0.127 | 6.16 | 693 | 289 | 18 |
| A22 | Alb | 48.38 | 9.45 | Beech | 0.302 | 0.175 | 5.875 | 542 | 409 | 49 |
| A33 | Alb | 48.39 | 9.49 | Spruce | 0.338 | 0.528 | 5.39 | 681 | 305 | 14 |
| A34 | Alb | 48.39 | 9.5 | Spruce | 0.267 | 0.518 | 4.74 | 626 | 353 | 23 |
| A42 | Alb | 48.4 | 9.45 | Beech | 0.414 | 0.241 | 6.285 | 504 | 386 | 110 |
| H02 | Hainich | 51.21 | 10.37 | Spruce | 0.21 | 0.387 | 5.225 | 308 | 600 | 92 |
| H03 | Hainich | 51.27 | 10.31 | Spruce | 0.253 | 0.508 | 4.85 | 409 | 549 | 43 |
| H07 | Hainich | 51.13 | 10.39 | Beech | 0.34 | 0.18 | 4.16 | 197 | 714 | 88 |
| H08 | Hainich | 51.36 | 10.52 | Beech | 0.375 | 0.195 | 5.275 | 227 | 716 | 58 |
| H09 | Hainich | 51.13 | 10.38 | Beech | 0.365 | 0.188 | 4.28 | 287 | 622 | 92 |
| H10 | Hainich | 51.09 | 10.46 | Beech | 0.101 | 0.055 | 4.655 | 447 | 503 | 53 |
| H11 | Hainich | 51.1 | 10.4 | Beech | 0 | 0.002 | 5.01 | 414 | 551 | 36 |
| H12 | Hainich | 51.1 | 10.46 | Beech | 0.137 | 0.069 | 3.985 | 168 | 713 | 117 |
| H13 | Hainich | 51.24 | 10.31 | Spruce | 0.177 | 0.389 | 6.52 | 510 | 444 | 48 |
| S01 | Schorfheide | 52.9 | 13.85 | Pine | 0.42 | 0.351 | 3.64 | 37 | 81 | 882 |
| S06 | Schorfheide | 52.91 | 13.84 | Beech | 0.573 | 0.319 | 3.665 | 49 | 77 | 874 |
| S07 | Schorfheide | 53.11 | 13.69 | Beech | 0.146 | 0.082 | 3.73 | 16 | 124 | 860 |
| S08 | Schorfheide | 53.19 | 13.93 | Beech | 0.087 | 0.059 | 3.38 | 47 | 198 | 755 |
| S09 | Schorfheide | 53.04 | 13.81 | Beech | 0 | 0.017 | 3.555 | 0 | 58 | 942 |
| S16 | Schorfheide | 53.08 | 13.86 | Pine | 0.433 | 0.335 | 3.57 | 30 | 108 | 862 |
| S40 | Schorfheide | 52.91 | 13.86 | Beech | 0.439 | 0.25 | 3.68 | 63 | 71 | 866 |
| S43 | Schorfheide | 52.9 | 13.93 | Beech | 0.401 | 0.229 | 3.715 | 69 | 121 | 810 |
| S48 | Schorfheide | 53.05 | 13.84 | Beech | 0.104 | 0.07 | 3.705 | 85 | 190 | 725 |
