# Supplementary material for: Biological Soil Crust From Mesic Forests Promote a Specific Bacteria Community
Source: Front Microbiol. 2022 Mar 16;13:769767. doi: 10.3389/fmicb.2022.769767 (PMC8966483; doi:10.3389/fmicb.2022.769767)
Supplement: Supplementary file 2 [file Table_2.DOCX]

Supplementary Table 2. Abundance of bacteria phyla in each sampling site separated to biocrust and bulk soil. Significant differences were calculated by ANOVA followed by Tukey Post-Hoc test, differences are indicated by letters. Bacterial phyla were ordered by total abundance.

|  | biocrust | | | | | | bulk soil | | | | | |
| --- | --- | --- | --- | --- | --- | --- | --- | --- | --- | --- | --- | --- |
|  | Alb | | Hainich | | Schorfheide | | Alb | | Hainich | | Schorfheide | |
| Proteobacteria | 28133 | ^bc^ | 26672 | ^abc^ | 31250 | ^b^ | 22218 | ^acd^ | 21049 | ^ad^ | 18612 | ^d^ |
| Actinobacteria | 5608 | ^c^ | 15930 | ^ab^ | 12025 | ^ac^ | 11678 | ^ac^ | 13111 | ^a^ | 20581 | ^b^ |
| Acidobacteria | 11209 | ^a^ | 6399 | ^a^ | 8532 | ^a^ | 9567 | ^a^ | 9616 | ^a^ | 11460 | ^a^ |
| Planctomycetes | 760 | ^a^ | 838 | ^a^ | 1093 | ^a^ | 10900 | ^bc^ | 11479 | ^b^ | 8925 | ^c^ |
| Bacteroidetes | 8916 | ^a^ | 5763 | ^a^ | 8501 | ^a^ | 1338 | ^b^ | 1313 | ^b^ | 997 | ^b^ |
| Chloroflexi | 3747 | ^ab^ | 6156 | ^a^ | 1055 | ^b^ | 2478 | ^b^ | 2589 | ^ab^ | 1277 | ^b^ |
| Verrucomicrobia | 3958 | ^a^ | 2562 | ^abc^ | 1144 | ^b^ | 3534 | ^a^ | 2751 | ^ac^ | 1348 | ^bc^ |
| Gemmatimonadetes | 1522 | ^c^ | 742 | ^ab^ | 532 | ^a^ | 1072 | ^bc^ | 1251 | ^bc^ | 319 | ^a^ |
| Firmicutes | 457 | ^abd^ | 151 | ^a^ | 319 | ^ab^ | 1208 | ^bcd^ | 1325 | ^cd^ | 1579 | ^c^ |
| Nitrospirae | 1243 | ^a^ | 546 | ^a^ | 24 | ^a^ | 293 | ^a^ | 467 | ^a^ | 152 | ^a^ |
| WPS-2 | 398 | ^a^ | 139 | ^a^ | 659 | ^a^ | 95 | ^a^ | 72 | ^a^ | 429 | ^a^ |
| NA | 230 | ^ab^ | 252 | ^ab^ | 130 | ^a^ | 325 | ^b^ | 374 | ^b^ | 293 | ^ab^ |
| Patescibacteria | 174 | ^a^ | 343 | ^a^ | 1041 | ^b^ | 10 | ^a^ | 11 | ^a^ | 23 | ^a^ |
| Rokubacteria | 5 | ^a^ | 1 | ^a^ | 0 | ^a^ | 828 | ^b^ | 518 | ^b^ | 4 | ^a^ |
| Entotheonellaeota | 0 | ^a^ | 0 | ^a^ | 0 | ^a^ | 539 | ^b^ | 208 | ^ab^ | 2 | ^a^ |
| Latescibacteria | 16 | ^a^ | 0 | ^a^ | 0 | ^a^ | 276 | ^b^ | 249 | ^b^ | 1 | ^a^ |
| Chlamydiae | 21 | ^a^ | 11 | ^a^ | 3 | ^a^ | 153 | ^bc^ | 125 | ^b^ | 200 | ^c^ |
| Cyanobacteria | 31 | ^a^ | 27 | ^a^ | 141 | ^b^ | 43 | ^a^ | 42 | ^a^ | 188 | ^b^ |
| Armatimonadetes | 53 | ^ab^ | 41 | ^ab^ | 103 | ^a^ | 42 | ^ab^ | 29 | ^b^ | 69 | ^ab^ |
| Elusimicrobia | 67 | ^a^ | 78 | ^a^ | 76 | ^a^ | 14 | ^a^ | 28 | ^a^ | 66 | ^a^ |
| Fibrobacteres | 59 | ^a^ | 6 | ^a^ | 33 | ^a^ | 24 | ^a^ | 12 | ^a^ | 8 | ^a^ |
| Spirochaetes | 53 | ^b^ | 3 | ^a^ | 8 | ^ab^ | 21 | ^ab^ | 25 | ^ab^ | 12 | ^ab^ |
| FCPU426 | 12 | ^a^ | 16 | ^a^ | 14 | ^a^ | 8 | ^a^ | 15 | ^a^ | 55 | ^b^ |
| Dependentiae | 14 | ^ab^ | 16 | ^ab^ | 2 | ^a^ | 17 | ^ab^ | 14 | ^ab^ | 38 | ^b^ |
| Omnitrophicaeota | 3 | ^a^ | 5 | ^a^ | 7 | ^a^ | 5 | ^a^ | 10 | ^a^ | 45 | ^b^ |
| FBP | 1 | ^a^ | 3 | ^a^ | 15 | ^a^ | 5 | ^a^ | 6 | ^a^ | 9 | ^a^ |
| WS2 | 7 | ^a^ | 5 | ^a^ | 0 | ^a^ | 4 | ^a^ | 4 | ^a^ | 0 | ^a^ |
| GAL15 | 0 | ^a^ | 0 | ^a^ | 1 | ^a^ | 3 | ^a^ | 0 | ^a^ | 15 | ^b^ |
| BRC1 | 7 | ^a^ | 3 | ^a^ | 0 | ^a^ | 2 | ^a^ | 6 | ^a^ | 0 | ^a^ |
| WS4 | 3 | ^a^ | 0 | ^a^ | 0 | ^a^ | 0 | ^a^ | 5 | ^a^ | 0 | ^a^ |
| Kiritimatiellaeota | 0 | ^a^ | 0 | ^a^ | 0 | ^a^ | 5 | ^a^ | 2 | ^a^ | 0 | ^a^ |
| Hydrogenedentes | 1 | ^a^ | 0 | ^a^ | 0 | ^a^ | 2 | ^a^ | 1 | ^a^ | 0 | ^a^ |
| Calditrichaeota | 0 | ^a^ | 0 | ^a^ | 0 | ^a^ | 1 | ^a^ | 0 | ^a^ | 0 | ^a^ |
| Nitrospinae | 0 | ^b^ | 0 | ^a^ | 0 | ^a^ | 0 | ^a^ | 1 | ^a^ | 0 | ^a^ |

Supplementary Table 3. List of bacteria families that were significantly (p<0.05) more abundant (number of OTUs) in biocrust compared to bulk soil calculated by Ancom analyses

| bacteria family | number of OTUs | |
| --- | --- | --- |
|  | in biocrust | in bulk soil |
| Bdellovibrionaceae | 1091 | 355 |
| Blastocatellaceae | 853 | 80 |
| Burkholderiaceae | 10053 | 1453 |
| Caulobacteraceae | 10295 | 1557 |
| Chitinophagaceae | 11200 | 1976 |
| Cytophagaceae | 915 | 99 |
| Inquilinaceae | 529 | 19 |
| Methylophilaceae | 114 | 2 |
| Micrococcaceae | 1965 | 43 |
| Micropepsaceae | 3198 | 328 |
| Microscillaceae | 2722 | 293 |
| Nitrosomonadaceae | 3411 | 582 |
| Pseudomonadaceae | 682 | 168 |
| Rhodanobacteraceae | 2416 | 158 |
| Rhodobacteraceae | 655 | 60 |
| Sphingobacteriaceae | 4976 | 240 |
| Sphingomonadaceae | 11688 | 101 |
| Spirosomaceae | 36 | 1 |
| Streptomycetaceae | 1088 | 700 |
| Xanthomonadaceae | 1001 | 72 |
