# Supplementary figures and images for: Biological Soil Crust From Mesic Forests Promote a Specific Bacteria Community
Source: Front Microbiol. 2022 Mar 16;13:769767. doi: 10.3389/fmicb.2022.769767 (PMC8966483; doi:10.3389/fmicb.2022.769767)

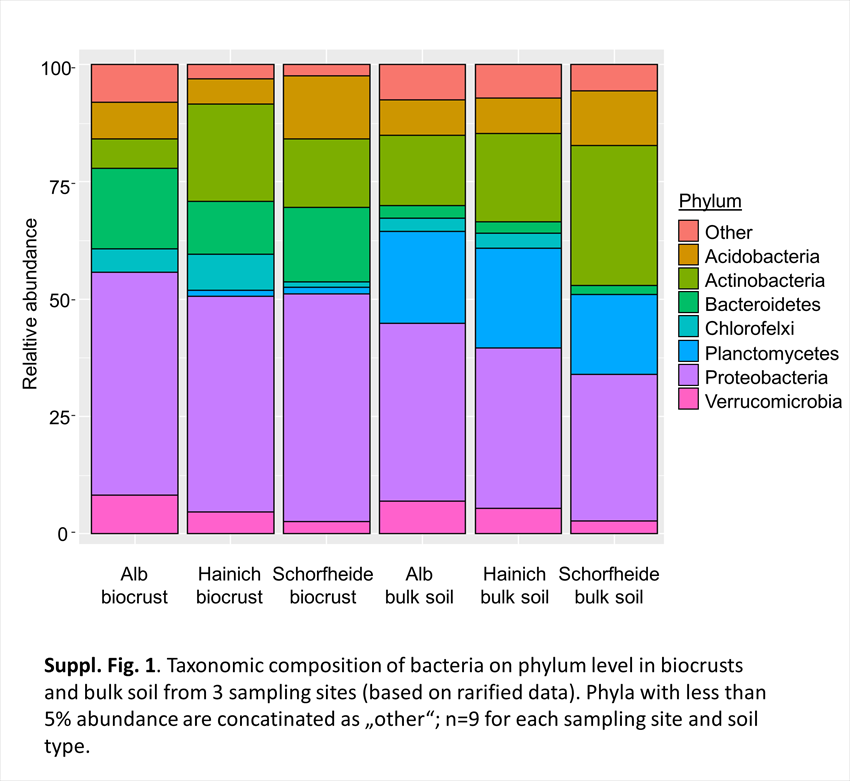

Supplement: Supplementary file 3 [file Image_1.TIF]
